# Regulatory Role of Nano-Curcumin against Tartrazine-Induced Oxidative Stress, Apoptosis-Related Genes Expression, and Genotoxicity in Rats

**DOI:** 10.3390/molecules25245801

**Published:** 2020-12-09

**Authors:** Gaber E. El-Desoky, Saikh M. Wabaidur, Zeid A. AlOthman, Mohamed A. Habila

**Affiliations:** Department of Chemistry, College of Science, King Saud University, Riyadh 11451, Saudi Arabia; swabaidur@ksu.edu.sa (S.M.W.); zaothman@ksu.edu.sa (Z.A.A.); mhabila@ksu.edu.sa (M.A.H.)

**Keywords:** nano-CUR, tartrazine, oxidative stress, antioxidant enzymes, apoptosis-related genes, genotoxcicity, experimintal rats

## Abstract

The present study evaluates the regulatory effect of Nano-Curcumin (Nano-CUR) against tartrazine (TZ)-induced injuries on apoptosis-related gene expression (i.e., p^53^, CASP-3 and CASP-9), antioxidant status, and DNA damages in bone marrow in treated rats. Male rats were arbitrarily separated into five groups, and each group was comprised of 10 rats each. The 1st group served as control (G1). The 2nd group ingested 7.5 mg TZ/kg. b.w. (body weight). The 3rd group ingested Nano-CUR 1 g/kg b.w. The 4th and 5th groups were respectively administered with (1 g Nano-CUR + 7.5 mg TZ/kg. b.w.) and (2 g Nano-CUR + 7.5 mg TZ/kg. b.w.). At the end of the experiment, blood samples, livers, and kidneys were collected. Livers and kidneys were homogenized and used for the analysis of reduced glutathione, malonaldhyde, total antioxidant capacity, lipid peroxide antioxidant enzyme activities, apoptosis-related gene expression, and genotoxicity by comit test. The ingestion of TZ for 50 days resulted in significant decreases in body, and kidney weights in rats and a relative increase in the liver weight compared to control. In contrast, the ingestion of Nano-CUR with TZ remarkably upgraded the body weight and relative liver weight compared to the normal range in the control. Aditionally, TZ ingestion in rats increased the oxidative stress biomarkers lipid peroxide (LPO) and malonaldehyde (MDA) significantly, whereas it decreased the reduced glutathione (GSH) levels and total antioxidant capacity (TAC). Similarly, the levels of glutathione peroxidase (GPx), superoxide dismutase (SOD), and catalase (CAT) significantly deteriorated in response to TZ ingestion. Moreover, the results revealed a remarkable up-regulation in the level of expression for the three examined genes, including p^53^, CASP-3, and CASP-9 in TZ-ingested rats compared to the control. On the other hand, the comet assay result indicates that the ingestion of TZ induced DNA damage in bone marrow. Notably, the administration of Nano-CUR protected the kidney and liver of TZ-ingested rats as evidenced by a significant elevation in all antioxidant activities of tested enzymes (i.e, SOD, GPx, and CAT), vital recovery in GSH and TAC levels, and a statistical decrease in LPO and MDA compared to TZ-ingested rats. Interestingly, the ingestion of rats with TZ modulates the observed up-regulation in the level of expression for the chosen genes, indicating the interfering role in the signaling transduction process of TZ-mediated poisoning. The results indicate that the administration of Nano-CUR may protect against TZ-induced DNA damage in bone marrow. According to the results, Nano-CUR exerted a potential protective effect against oxidative stress, DNA damage, and the up-regulation of apoptosis-related genes induced by TZ ingested to rats.

## 1. Introduction

Additives are normally added to foodstuffs to improve their flavor, color, texture, taste, and conservation [1]. Artificial food colors are large category of food additive used in food after approval by the Food Drug Administration (FDA) [2]. Color is a vital constituent of food that makes food look brighter, attractive, and informative, as it helps the consumers identify their desired food products [3]. Food dyes occupy an important place in the class of essential additives for the food industry in the conquest of markets. Among the most commonly used dyes by the food industry are those that contain the AZO group (–N=N–) including tartrazine (TZ), sunset yellow, and bordeaux red. Tartrazine (known as E102 or FD & C yellow 5, color index C.I-19140) is a synthetic lemon azo dye utilized as a food coloring agent that is derived from coal tar [4]. A regulated concentration policy for various dyes in foods, cosmetics, and drug has been emphasized and stated [5]. The admissible daily intake (ADI) of TZ for human is 0–7.5 mg/kg b.w. [6].

Several marketed food products contain TZ including cotton candy, flavored chips, soft drinks, cake mixer, cereals (corn flakes and muesli), sauces, soups, some rices, candies, ice cream, chewing gum, jam, marzipan, and jelly. In addition, some consumable products include TZ, which are cosmetics, soaps, shampoos and hair products. In addition, there are some pharmaceutical formulation containing TZ including vitamins, anti-acids, medicinal capsules, and specific prescription drugs [7]. Synthetic dyes have shown controversial effects on the health of humans. They exhibit detrimental effects on human lymphocytes interacting with genetic materials such as DNA [8]. Moreover, other studies have proposed that azo dyes are not carcinogenic and harmful to humans [9,10]. Therefore, the Food and Drug Administration (FDA) and World Health Organization (WHO) have frequently revised the safety regulations for dyes used as food color [6]. However, azo dyes including TZ, when consumed by rats for prolonged periods, can cause adverse effects, such as stomach inflammation and the amplification of lymphocyte and eosinophil counts [11]. Determinal effects of azo dyes such as bladder cancer were reported for workers exposed to benzidine-based dyes. The catabolism of azo dyes by mammalian gut microflora can produce aromatic amines, which might significantly be carcinogenic or mutagenic toward animals [12]. It was reported that different doses of TZ encourage hepatic and kidney pathological changes depending on concentration, age, genetic predisposition, nutrition, and extent of exposure to a particular dose [13]. One of the vital mechanisms of concern in azo dyes-induced toxicity is oxidative stress, since azo dyes exposure upsets the balance between the prooxidant and antioxidant system and produces excessive reactive oxygen species (ROS), which oxidizes the cellular components and results in DNA or protein damage and cells apoptosis [14]. The comet assay result of Latifa et al. [15] revealed that TZ caused DNA damage in leucocytes as well. This genotoxic effect is perhaps due to the direct contact between TZ and nuclear DNA [16]. In addition, TZ can induce chromosomal aberrations in fibroblast cells of various Muntiacus muntjac [17]. Hassan [18] in his research work also discovered that the ingestion of a daily dose of TZ (7.5 and 15 mg/kg b.w.) for a period of seven weeks led to DNA damage of the kidney and liver.

Hence, considering the oxidative damage of azo dye in various body organs, there has been a large amount of attention on the application of natural and active dietary phytochemical colors for coloring foods individually or with synthetic dyes. Curcumin, a promising hydrophobic polyphenolic compound, is a yellow pigment extracted from the rhizome of curcuma longa (turmeric). As a natural phenolic compound, it has possessed various health benefits such as antioxidant, anti-inflammatory, anticancer, antimicrobial, anti-Alzheimer, and wound-healing [19]. Curcumin has shown a wide range of therapeutic effects and pharmacological application [20,21,22]; the usage of curcumin in coloring food with or without synthetic colors strengthens the cell antioxidants and protects from synthetic dyes-induced oxidative damage. However, this application of curcumin has been quite limited, because of its extremely low solubility in water media, poor absorption in the gastrointestinal environment, and very low oral bioavailability. It has been reported that curcumin can be solubilized only less than 0.6 µg/mL in pure water and 1 mg/mL in ethanol [23]. In order to overcome these limitations, many studies have focused on improving its solubility with or without organic solvent because it could not be applied to the food system when it uses any organic solvent in whole processing. One method used only mechanical techniques such as high-pressure homogenization (HPH) to improve the curcumin solubility; another technique used ultrasonic homogenization to make curcumin nano-suspensions in water because it is an easy and fast process to make smaller solid dispersion by breaking the intermolecular as well, as it does not need any organic solvent [24]. The conversion of curcumin (CUR) to Nano-CURcumin (Nano-CUR) has a lot advantages: (I) increased solubility and stability in water; (II) enhanced oral bioavailability and absorption rate [19]; (III) widespread usage of Nano-CUR as a natural color with or without synthetic colors for coloring food, cosmetics, and drugs; (IV) governing the oxidative stress in different processed foods, and (V) protection in living cells against ROS-mediated oxidative damage.

Thereby, the purpose of the present study was to prepare Nano-CUR and apply it in combination with TZ to produce a new blend of yellow color used for coloring food, drug, and cosmetics. Additionally, it also includes an evaluation of the oxidative stress biomarkers of TZ on animals and the TZ-mediated expression level of selected apoptosis-related marker genes (i.e., TP^53^, caspase-3, caspase-9) via quantitative reverse-transcriptase-PCR (qRT-PCR). The investigation on the protective effect of Nano-CUR in ameliorating the oxidative damage of hepatic and kidney injuries in male rats fed a diet containing TZ has been carried out as well. Moreover, the impact of Nano-CUR, co-administration on TZ-mediated expression level of selected apoptosis-related marker genes (TP^53^, caspase 3, caspase 9) via quantitative-transcriptase (qRT-PCR) and the genotoxic effects of TZ on bone marrow cells and the regulatory effects of Nano-CUR on TZ-treated rats has been investigated and evaluted.

## 2. Results

### 2.1. Morphological Characterization of Nano-CUR

**Figure 1:**
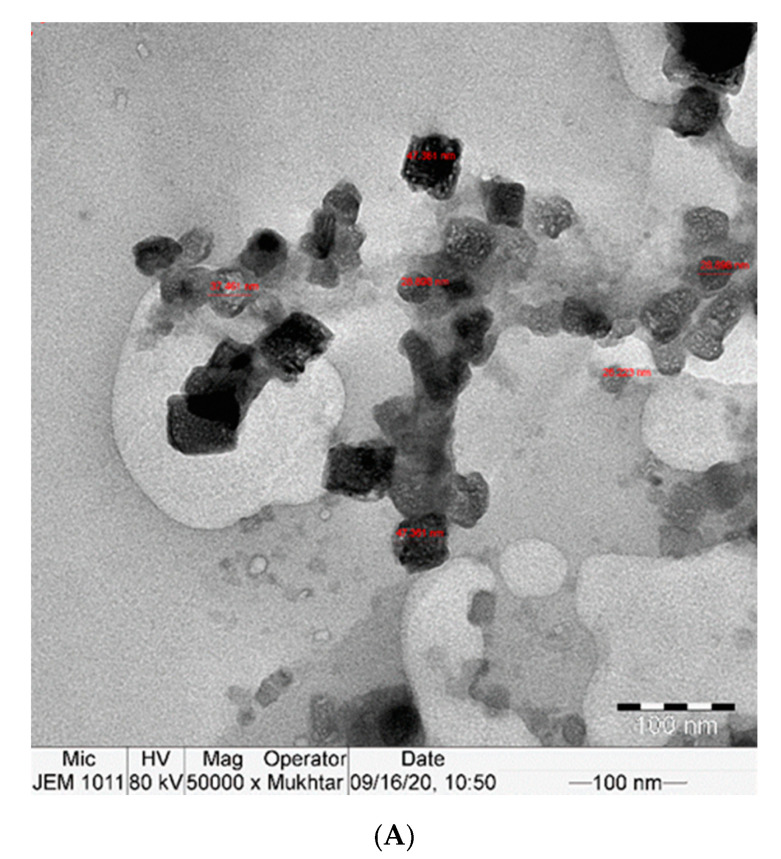

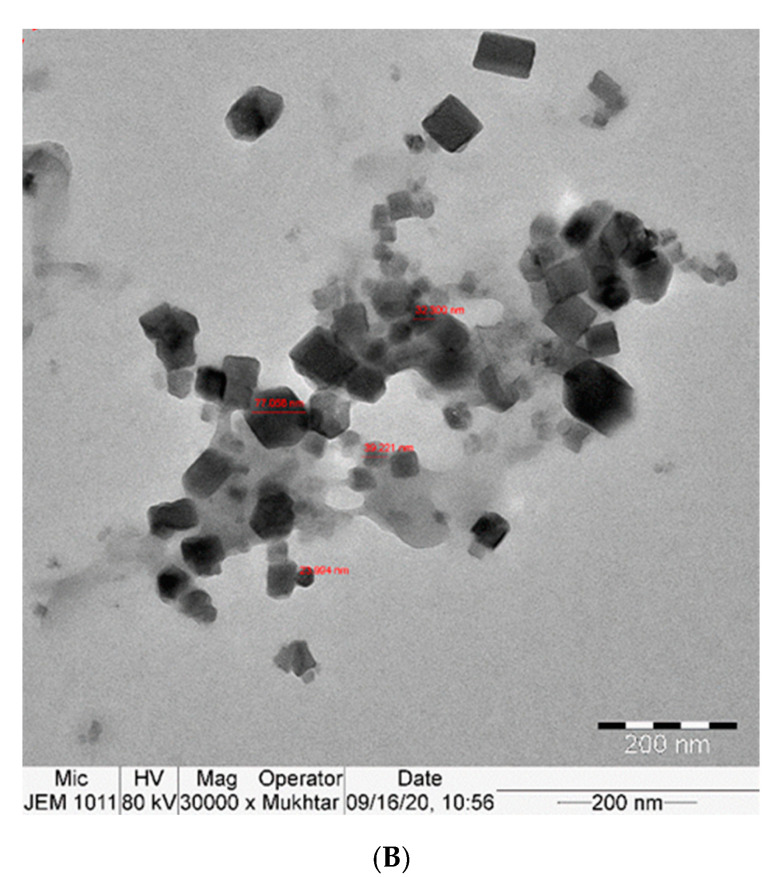
(**A**,**B**): TEM image of Nano-CURcumin (Nano-CUR) showing particles size 26–78 nm.

The TEM examination showed the uniform cubic shape of curcumin nanoparticles with a particle size between approximately 26 and 70 nm. The prepared curcumin nanoparticles were formed with a stable surface charge, which led to well-separated nanoparticles in the whole sample, as shown in Figure 1A,B.

### 2.2. Effect of Nano-CUR on Body Weight Relative Liver and Kidney Weights

After 50 days of the experiment, no death was recorded in all experimental animals. TZ treatment (G2) caused significant (*p* < 0.01) decreases in body weight, body weight gains, and relative kidney weight compared to control G1. In contrast, relative liver weight significantly increased in TZ-treated rats compared to control G1 (Table 1).

The co-ingestion of Nano-CUR and TZ to rat groups (4 and 5) showed significant increases in body weight, body weight gain, and relative kidney weight but significant decreases in relative liver weight compared to TZ-treated rats (G2); the increase and decrease values were Nano-CUR dose-dependent (Table 1). The ingestion of Nano-CUR alone to the rats of group 3 had significant (*p* < 0.01) increases in body weight, body weight gains, and relative kidney weight, whereas relative liver weights showed significant decrease values compared to TZ-treated rats. No obvious changes in body weight, body weight gain, relative kidney weight, and relative liver weight were observed between control (G1) and Nano-CUR groups (3, 4, and 5) (Table 1).

### 2.3. Effect of Nano-CUR on LPO, MDA, GSH, and TAC as Oxidative Biomarkers

As shown in Table 2, TZ ingestion to rats (G2) significantly (*p* < 0.01) amplified blood LPO and MDA in kidney and liver tissues; these upsurges were by 21.3%, 29.7%, and 27.5% respectively relative to control (G1). The co-ingestion of (Nano-CUR plus TZ) to rat groups 4 and 5 blocked these enhanced productions by 25.3%, 15.4%, and 17.0% for group 4 and by 29.2%, 30.3% and 30.7% for group 5 respectively, relative to TZ-ingested rats (G2). A comprehensive normalization in blood LPO and MDA contents in kidney and liver were evident in groups 4 and 5 compared to its normal levels in control G1. Moreover, significant (*p* < 0.01) decreases in blood TCA and reduced glutathione (GSH) levels in liver and kidney tissues as a TZ ingestion function (G2) were noticed as follows: 14.3%, 33.2%, and 23.1% compared to control (G1). The co-ingestion of Nano-CUR and TZ (G4 and G5) recovered the contents of blood TAC and GSH in liver and kidneys by 43.9%, 15.4%, and 28.2% for group 4 and by 31.3%, 30.1%, and 31.2% for group 5 respectively relative to TZ-ingested rats (G2).

The co-ingestion of nano-CUR and TZ to rats (G4 and G5) normalized the levels of blood TAC and GSH in kidney and liver compared to its values in the control group (G1). The ingestion of Nano-CUR alone to rats in group 3 exhibited positive effects on blood LPO and TAC as well as GSH and MDA contents in liver and kidneys compared to those values in the control group (G1).

### 2.4. Nano-CUR Activities on Antioxidant Biomarker Enzymes (SOD, CAT, and GPx) in TZ-Treated Rats

The results listed in Table 3 specified that the kidneys and liver of TZ-ingested rats (G2) had significantly (*p* < 0.01) reduced the activities of important antioxidant enzymes including SOD, CAT, and GPx relative to control (G1). These reductions were recorded as follows: 34.4%, 25.1%, and 34.1% for livers and 33.0%, 29.3%, and 33.1% for kidneys respectively compared to control (G1). In rats co-administrated with (Nano-CUR plus TZ) (G4 and G5), a significant (*p* < 0.01) recovery on the activities of CAT, SOD, and GPx were by 33.3%, 44.9%, and 48.2%, respectively in the liver and 39.9%, 39.5%, and 48.7%, respectively in kidneys of G4 compared to TZ-ingested rats (G2). Whereas these recoveries were by 50.8%, 33.7%, and 51.5% in liver and by 46.0%, 39.9%, and 49.9% in kidney respectively in group 5 (G5) relative to TZ-treated rats (G2). Conversely, an ingestion of Nano-CUR individually to rats (G3) did not show significant changes in the performances of these enzymes either in the kidney or liver relative to control (G1), whereas there were noteworthy (*p* < 0.01) upsurges in the performance of these enzymes in the kidney and liver relative to TZ-ingested rats (G2).

### 2.5. Influence of Nano-CUR on Total Proteins

Total proteins in kidneys and liver of rats ingested with TZ (G2) were intensely (*p* < 0.01) reduced by 23.7% and 37.8%, respectively relative to control (G1). The co-ingestion of Nano-CUR with TZ to rats (G4 and G5) showed significant increases in total protein in the liver and kidneys by 51.6% and 29.4% respectively in group 4 (G4), whereas these increases were by 59.0% and 31.0% respectively in group 5 (G5) compared to TZ-ingested rats (G2). The ingestion of Nano-CUR without or with TZ (G3, G4, and G5) did not have any significant changes on total protein contents in liver and kidneys relative to control (G1), as shown in Table 3.

### 2.6. Influence of Nano-CUR on the Transcriptional Activity of Stress and Apoptosis-Related Genes

**Figure 2:**
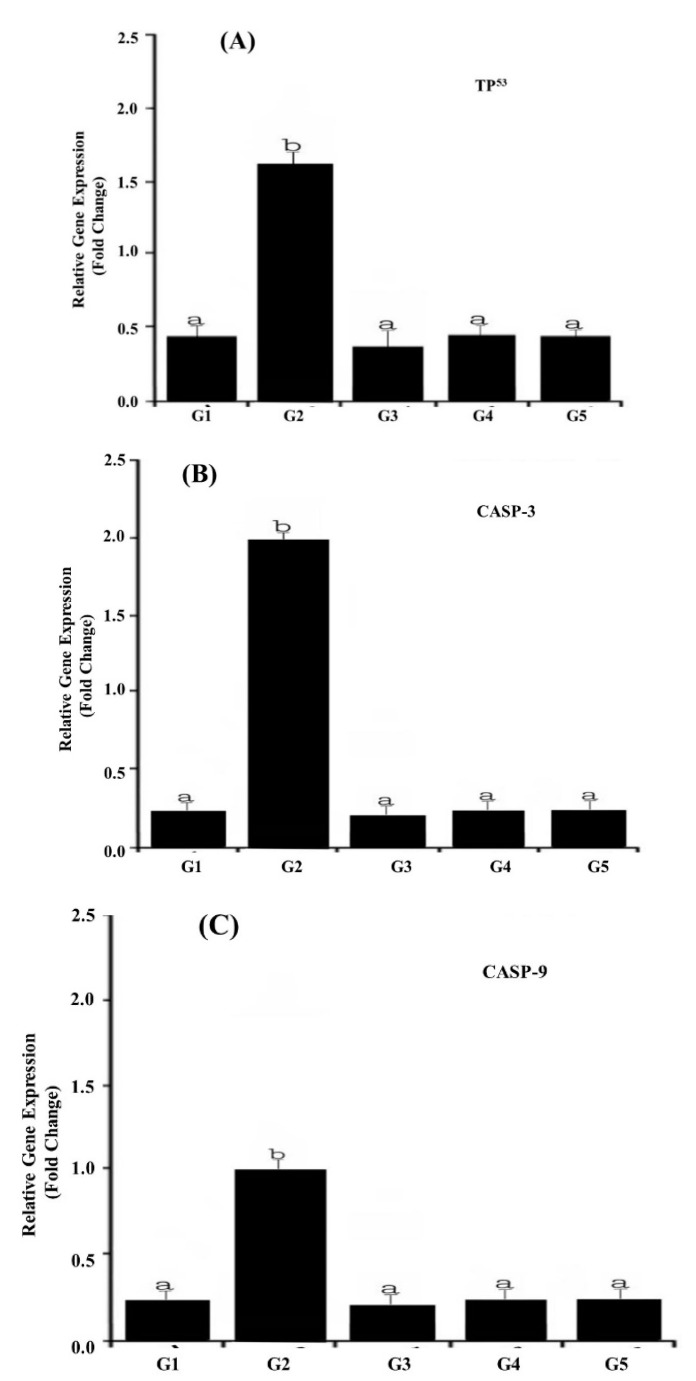
LightCycler^®^480 real-time PCR analysis of apoptosis-related genes in TZ-ingested rats and Nano-CUR ingested rats. The PCR analysis was performed using isolated and reverse transcribed RNA with primers listed in Table 4. CASP-2 methods were applied for relative expression determination using PCR efficiencies, which were determined with the standard curve of each run. The data point denotes the results obtained from three individual batches of cDNA made for each stage or tissue (**A**) TP^53^ gene expressions, (**B**) CASP-3 gene expression, and (**C**) CASP-9 gene expression of target genes, which were normalized to the reference glyceraldehyde-3-phosphate dehydrogenase (GAPDH) gene and indicated as mean ± S.E. The different alphabet letters specify different mean values at *p* < 0.05 by a paired Student *t*-test.

The transcriptional activity of three key stress and apoptosis-related genes including TP^53^, CASP-3, and CASP-9 in response to TZ-ingested rats is shown in Figure 2A–C. Our results revealed that the ingestion of TZ to rats (G2) significantly (*p* < 0.01) up-regulated the expression of apoptosis-related genes i.e., TP^53^, CASP-3, and CASP-9 (Figure 2A–C) compared with the control G1.

The co-ingestion of (Nano-CUR with TZ) to rats (G4 and G5) had profound modulatory effects on the observed TZ-mediated up-regulation of expression in TP^53^ (Figure 2A), CASP-3 (Figure 2B), and CASP-9 (Figure 2C) genes, leading to their transcriptional resistance. In response to TZ, the Nano-CUR supplementation adversely influenced the expression level of all three genes, which was approximately normalized to its level in the control group (G1).

The ingestion of Nano-CUR to rats alone (G3) showed no noteworthy variations in the transcriptional activity of TP^53^, CASP-3, and CASP-9 genes relative to those in the control (G1).

### 2.7. Genotoxicity Results

**Figure 3:**
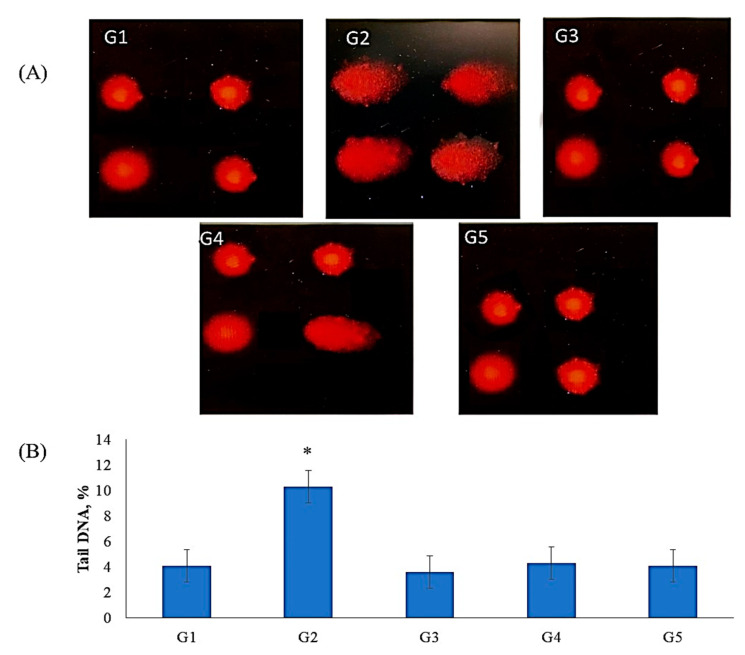
The ameliorating effect of Nano-CUR on genotoxicity induced by TZ-ingested rats: (**A**) Fluorescence micrograph of bone marrow cells nuclei after the comet assay. The untreated rats (G1) (control) show no detectable DNA damage. The nuclei of TZ-ingested rats (G2*) appeared damaged. The nuclei of Nano-CUR ingested rats (G3) appeared to have no detectable DNA damage as control. The nuclei of (1 g Nano-CUR + 7.5 mg TZ-mix)-ingested rats appeared to have slight damage, the nuclei of (2 g Nano-CUR + 7.5 mg TZ-mix)-ingested rats appeared to have no detectable DNA damage. (**B**) Bar graph showing the tail DNA damage percentage in nuclei of bone marrow of all treated rats subjected to comet assay. Data are mean ± S.D. (*n* = 10, *p* < 0.05).

Comet assay results indicated that TZ owns a genotoxic influence in bone marrow cells of TZ-ingested rats (Figure 3A,B). This genotoxic effect was significantly (*p* < 0.01) increased in the DNA percentage of comet tails in the nuclei of bone marrow cells of TZ-ingested rats compared to the control (G1). The ingestion of Nano-CUR with TZ to rats (G4 and G5) suppresses the increases in DNA percentage of comet tails in the nuclei of bone marrow cells relative to tartrazine-ingested rats (G2). The suppression was higher in group 5 rats that ingested (2 g of Nano-CUR + 7.5 mg of TZ/kg b.w.) than that in group 4, which ingested (1 g of Nano-CUR + 7.5 mg TZ/kg b.w.) relative to group 2 and normalized to its level in control (G1). The ingestion of Nano-CUR alone to rats group3 (G3) showed no significant (*p* < 0.01) changes in the DNA comet tail in the nuclei of bone marrow relative to control (G1), whereas there was a significant (*p* < 0.05) decrease in the DNA tails in the comet assay of bone marrow relative to TZ-ingested rats (G2), as shown in Figure 3A,B.

## 3. Discussion

Body mass and relative liver masses are good indicators of toxicity and pathology [25]. The remarkable reductions in body weight gain and relative kidney weight were noticed, whereas the relative liver weights of TZ-ingested rats were found to be increased relative to the control. This might be due to TZ ingestion, which reduces the palatability of food or otherwise results in avoidance. Furthermore, TZ ingestion might result in the production of free radicals, which results in oxidative stress and consequently causes metabolic disorders and body weight losses. Ezeuko vitalis and his group [25] stated that weight loss or reduced weight gain and increased liver weight are considered to be a sign of toxicity. In TZ-treated rats, the possible explanation for increasing liver weight may be due to Tz toxicity, which causes inflammation and enlargement, thus increasing its weight. In contrast, growth retardation might be due to the TZ dye’s disturbance effect that leads to a decrease in the population of intestinal bacteria and as well as a hindered food absorption capacity of the intestinal surface. Chung et al. [26] reported that azo dye can be reduced to aromatic amines, for example, sulfanilic acid in the presence of intestinal microbial azo reductase. Some of those reduced products are highly carcinogenic and hinder the digestion of absorption of food.

The addition of CUR in the form of Nano-CUR to TZ-ingested rats increased the antioxidant capacities or free radicals scavenging power, which might improve the digestion, adsorption, and metabolism of food. In addition, Hassan et al. [27] described that the relative liver weight is highly involved in judging the pathological condition of the liver. Therefore, a drop in the relative liver weight and upsurge of weight gain in Nano-CUR-treated rats indicated an improvement of the liver to return to the normal conditions.

In the present study, MDA and LPO in TZ-ingested rats compared to control indicate that TZ elevates oxidative stress in the body, which may be due to the formation of ROS leading to lipid peroxidation, which is estimated by an increase in the thiobarbituric acid (TBARS) level [28]. Moreover, TZ may enhance peroxidation, interacting directly with the cellular plasma membrane [28]. In the same respect, TZ is metabolized inside the body into aromatic amines interacting with intestinal microflora. The active amino groups interact with nitrate and nitrite-containing foods and produce ROS as part of their metabolism [10]. Similarly, in TZ-treated rats, lower concentrations of GSH and TAC were noticed compared to the control, which may be due to the formation of ROS, and the metabolism of TZ in small intestine produces genotoxic components including hydrogen peroxide, hydroxyl radicals, and superoxide anion, resulting in oxidative stress. The decreased TAC levels in blood and GSH content in the kidney and liver of TZ-ingested rats was attributed to an increase in the free radical productions or impaired antioxidant machinery, resultiing in amplified oxidative stress. The elaborated battery of the enzymatic defense system consisted of SOD, CAT, and GPx; or, the non-enzymatic system by the scaving action of GSH can be used for the detoxification of ROS, while the activity of glutathione S-transferase (GST) can be applied for the detoxification of organic peroxides [29].

The modulation of these enzymes activities and GSH levels plays a primary role balancing the redox status by reducing ROS and forming peroxides in the organism and xenobiotic detoxifications [30]. In addition, a significant reduction in the activity of key antioxidant enzymes including SOD, CAT, and GPx was noticed in TZ-ingested rats. Basically, SOD, an enzymatic defensive system allowing the dismutation of superoxide ion (O_2_^●^^−^) in H_2_O_2_, and their accumulation was avoided by the CAT/GPx system, transforming it into water and molecular oxygen (O_2_) or oxidized glutathione (GSSH), respectively [31]. The most important metabolic role of GPx is the antioxidant enzyme formation [29]. GPx can prevent oxidative damage of the cell membrane and protect cells against cleavage by ROS. It also protects membrane lipids from oxidation generatin peroxide and allows the regeneration of membrane lipid molecules via recylation [29]. Thus, GPx may resist the toxic influences of free radicals and reduce the production of the reactive metabolites induced by TZ. When such systems fail or become suppressed, an overproduction of O_2_^●^^−^ and H_2_O_2_ gives rise to the highly toxic hydroxyl radical formation (OH^●^).

It is well known that xenobiotics can induce the production of mitochondrial superoxide radicals [32], and additionally, SOD inhibits the formation of O_2_^●^^−^ in the cell, which can reach unsafe levels. Superoxide radical is a powerful inhibitor of CAT [33]. Therefore, in this study, the observed depletion of antioxidant enzyme activity (Table 3) could be caused by a direct influence on the enzyme by TZ-induced ROS generation, diminution of the enzyme substrates, and down-regulation of translation and transcription processes. Nano-CUR ingestion without (G3) or with TZ (G4 and G5) successfully normalized the levels of MDA, LPO, GSH, and TAC with respect to control (G1). Similarly, it significantly reduced MDA and LPO levels relative to TZ-treated rats (G2). Therefore, GSH and TAC levels were significantly hiked in Nano-CUR-ingested rats compared with the TZ-ingested rats (G2). These results suggest that Nano-CUR possesses a powerful antioxidant activity against TZ-induced oxidative damage. This makes Nano-CUR able to scavenge oxygen from radicals, resulting in an increment in intracellular GSH levels, and as a result, it effectively controls the levels of lipid peroxidation. Zheng et al. [34], in their research study, suggested CUR as a efficient inducer of the de novo synthesis of GSH. Similarly, the ingestion of Nano-CUR potentially increased the activity of SOD, CAT, and GPx enzymes relative to TZ-treated rats. In contrast, Nano-CUR helped normalize the activities of these antioxidant enzymes relative to the control (G1), which indicated that Nano-CUR was able to block the adverse consequences of ROS formed by the metabolism of TZ and up-regulate the translation and transcription processes of the enzymes. These findings are in agreement with the previously published studies, reporting that CUR is a powerful antioxidant that attends to oxidative stress in experimental models [35].

According to the literature, the beneficial activities of CUR are associated with its structure. Two phenolic rings and beta-ketone sections in the chemical skeleton of CUR are responsible for its high antioxidant properties [36]. However, studies have indicated that the protective influence of CUR is less important than that of its Nano form due to its considerably low solubility and bioavailability [37]. On the other hand, more recent findings have shown that Nano-CUR could be used to address some of the limitations of CUR (e.g., low solubility and bioavailability) [38]. The obtained results of the present research study also confirmed the better outcomes of Nano-CUR compared to curcumin alone, as the nano-CURcumin increase its solubiliuty and bioavailability in the body [39].

Several in vivo studies have described that azo dyes are insecure, as they cause tumors in the urinary bladder and liver of rats [40]. In addition, Patterson and Butler [41] have mentioned in their research finding that azo dyes can induce chromosomal aberrations in mammalian cells, resulting in DNA damage in the colon of mice [13]. Therefore, we have observed the impact of TZ treatment on the selected apoptosis-related genes expression, namely, CASP-3, CASP-9, and TP^53^. Our results discovered that TZ ingestion to rats for a period of 50 days showed a significant up-regulation of expression of apoptosis-related genes compared with the control (Figure 2A–C). Consequently, apoptotic cell death happens. Apoptosis has been associated with DNA damage or mitochondrial or cell-cycle activation. The classical apoptotic death process produces a permeability transition pore complex (PTPC) that allows the release of cytochrome-C, endonuclease G, apoptosis-inducing factor (AIF), and the second mitochondria-derived activator of Caspase, Smac (DIABLO) [42]. The release of cytochrome-C protein into the cytoplasm induces the formation of the apoptosome complex, which actively takes part in the activation of initiator procaspase-9 [43]. Subsequently, the caspase cascade is successively amplified through the activation of executioner caspase (e.g., caspase-3, caspase-6, and caspase-7) [44].

The obtained results are in line with the earlier literature that reported that apoptotic cell death is caused by a series of proteases (caspases), which are activated during the apoptotic processes and categorized as initiator caspases (e.g., CASPS-8, CASPS-9, and CASPS-10) and effector caspases (e.g., CASPS-3, CASPS-6, and CASPS-7) [45]. The activation of initiator caspases leads to protein PARP-1. PARP-1 causes DNA to break down, and consequently, apoptotic cell death occurs.

**Figure 4:**
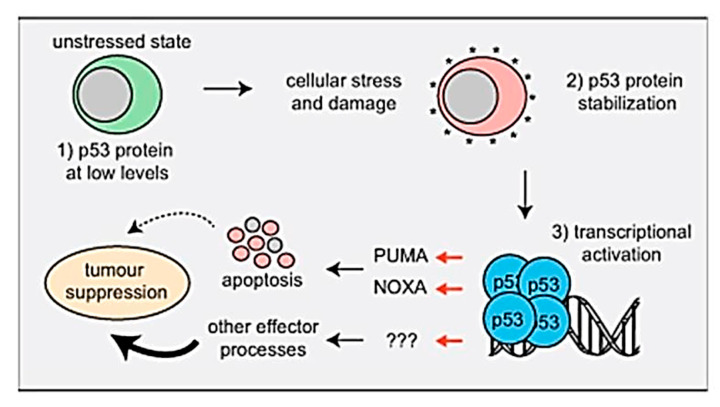
The mechanistic pathway of P^53^-induced cell death and its effect on TP^53^-mediated tumor suppression.

Furthermore, the expression of the up-regulated selected apoptosis-related gene TP^53^ related to TZ-treated animals was confirmed by the results of Aubrey et al. [46], who discuss the mechanisms by which P^53^-induced cell death occurs and how this affects P^53^-mediated tumor suppression genes and the malignant cells response during the anticancer therapy, as shown in the following Figure 4 and facts about P^53^.

## 4. Facts

- TP^53^ works as a critical tumor suppressor and is mutated in 50% of human cancers.
- In unstressed cells, the levels of the p^53^ protein are very small because the E3 ubiquitin ligase MDM2 targets it for proteasomal degradation.
- TP^53^ is activated in response to the activation of oncogenes and DNA damage stress stimuli.
- Activated p^53^ regulates the transcription of approximately 500 genes directly and many additional genes by indirect ways. Thereby, it controls diverse cellular procedures.
- Non-transformed cells induce apoptosis by P^53^ mostly by the direct transcriptional activation of the proapoptotic BH_3_-only proteins (PUMA and NOXA).
- A combined loss of the PUMA plus NOXA and cell cycles arrest/cell senescence (p^21^) is not responsible for spontaneous tumor growth.
- PUMA and NOXA-induced apoptosis is critical for the malignant cells death by anticancer medications that activate TP^53^, but other effectors contribute [46].

Similarly, the genotoxicity results of the comet assay indicate that TZ treatment caused DNA damage in bone marrow cells. This genotoxicity is probably associated with the direct interaction of TZ with nuclear DNA [16]. Therefore, the comet assay can be used as a specific test for detecting genotoxicity, and the results are not necessarily confounded by concomittant processes leading to apoptosis. The results show that apoptosis does not necessarily need to correlate or coincide with DNA damage observed with genotoxic substances in the comet assay.

These results of genotoxicities of TZ-treated rats were in good aggreeent with the findings of Mpountoukas et al. [8]. They demonstrated the toxicity of TZ at 0.02–8 mM in peripheral blood cells of humans in vitro. In addition, TZ has been found to be effective in inducing chromosomal aberration in fibroblast cells of Muntiacus muntjac [17]. In addition, the administration of TZ (7.5 mg and 15 mg/kg b.w.) on a daily basis for 7 weeks led to kidney and liver DNA damage.

Poul et al. [47] showed that the oral administration of TZ at a dose up to 2 g/kg b.w. did not induce genotoxic attritions in micronucleus test assay in mice. Alternatively, at a slightly higher dose than the recommended daily intake proposed by the joint FAO/WHO expert committee on food additives [6], TZ-induced DNA damage in comet assay was observed in cells from the colon of mice due to TZ induction [48].

The ingestion of Nano-CUR with TZ to rats G4 and G5 showed ameliorating effects on DNA damaged as detected in comet assay (Figure 3). The obtained findings clearly indicate that Nano-CUR ingestion significantly modulates the transcriptional activity of the primary apoptosis-related gene, which is indicative of an interfering defensive role in the TZ-mediated poisoning signaling transduction process. In combination, it may be recommended that the oxidative stress characteristic and poisoning of exposure to TZ may be partially enhanced by supplementation with Nano-CUR. This might be due to the potential antioxidant role of Nano-CUR and ROS neutralized capacity, whereby preventing cellular components and DNA damage leads to the enhancement of the apoptosis-related pathway [49]. In the same respect, El-Bahr [50] mentioned that the characteristic protective effect of CUR was via the expression of a gene subset, since it has been revealed to regulate the gene expression of insulin-like growth factor B-cell CLL/lymphomas and antioxidant enzymes in streptozotocin-induced diabetic rats. Thus, it was concluded that Nano-CUR plays an important role by acting against oxidative stress and genotoxicity induced by TZ ingestion. The obtained comet assay control group (G1) results range around 4% (Figure 3) for the experimental rats, which are at very low basal levels in mammalian cells. The lower results might be due to the adaptation of good experimental conditions of treated rats, such as air conditions, healthy rats, healthy diet, etc. [51]. These leads to the lower percentage values of the control group, and moreover, the successful application of the comet assay.

## 5. Materials and Methods

### 5.1. Chemicals

Tartrazine (TZ) (C.I. 19140 CAS No. 1934-21-0, MW 534.37; Synonyms: E 102, FD & C yellow 5, Food yellow 4), chemically known as trisodium 5-hydroxy-1-(4-sulfonatophenyl)-4-(*E*)-(4-sulfonatophenyl) diazenyl-1*H*-pyrazole-3-carboxylate, was purchased from Sigma-Aldrich Corp. (St. Louis, MO, USA). The manufacturer assured a purity of 86.7%. CUR was procured from Sigma-Aldrich chemical company (St. Louis, MO, USA).

### 5.2. Preparation of Nano-CUR

All applied chemicals were of analytical grade. Deionized water was used in all steps for the cleaning of glasses as well as for the preparation of curcumin nanoparticle suspension. The method described in [52,53,54] was followed after some modification, in which a certain weight of curcumin was added to a water/ethanol mixture and exposed to ultrasonic waves for 30 min. Then, the mixture was heated at 60˚C for 2 h to obtain the curcumin nanoparticles suspension. Then, the formed curcumin nanoparticles were collected by centrifuge followed by freeze-drying and kept until use. The shape and particle size of the prepared curcumin nanoparticles was observed via Transmission Electron Microscopy (TEM).

The morphology and particle size of the produced nano-curcumin were characterized by scanning electron microscope (model S-3400-N, Hitachi, Tokyo, Japan). The particles were fixed by a conductive adhesive tape on aluminum stubs and coated by a gold layer using a sputter coater. The particle size and distribution were measured using a particle size analyzer apparatus (Coulter Model LS 130, Coulter Electronics, company, Indianapolis, IN 46268, USA). The samples were suspended in deionized water and sonicated for 1 min by an ultrasound system (500 W, Vibra Cell, Sonics and Materials, Inc., Newtown, CT 06470, USA). Then, they were added into a transparent cell and located on the light pass. An X-ray diffraction analyzer (D8 FABLINE, Buker, Newtown, CT, USA) was utilized to characterize the crystalline pattern.

## 6. Experimental

### 6.1. Animals

Male, albino, Wister rats of weight between 220 and 230 g were obtained from the Animal House, King Saud University (Riyadh, Saudi Arabia). Rats were housed in polypropylene cages and allowed to adapt to the laboratory environment for seven days before the start of the experiments. The animals were kept under controlled conditions including 23 ± 1 °C temperature, 50 ± 15% humidity, and a photoperiod of 12 to 12 h light–dark cycles. Rats were provided a standard basal diet (fat 5%, carbohydrate 65%, protein 20.3%, fiber 5%, salt mixture 3.7% and vitamins mixture 1%) and water ad libitum. The current study was performed in the College of Pharmacy’s animal house (King Saud University). The handling and care of rats followed the Animal Ethical Committee (College of Pharmacy, King Saud University) KSU-REC 008E.

### 6.2. Expermintal Design

The rats were randomly divided into five different groups of 10 rats each, housed in polypropylene cages, and provided a standard basal food and water diet ad libitum. The groups were as follows:

- Group 1 (G1): Rats orally ingested 1 mL of distilled water daily for 50 days and used as control.
- Group 2 (G2): Rats orally ingested tartrazine (TZ) 7.5 mg/kg b.w. (prepared in 1 mL distilled water) daily for 50 days.
- Group 3 (G3): Rats orally ingested Nano-CUR 1 g/kg b.w. dissolved in 1 mL of distilled water daily for 50 days.
- Group 4 (G4): Rats orally ingested a mixture of (1 g Nano-CUR + 7.5 mg of TZ/kg b.w.) dissolved in 1 mL of distilled water daily for 50 days to understand if the increasing concentration of nano-CUR can cause any toxicity.
- Group 5 (G5): Animals orally ingested a mixture of (2 g Nano-CUR + 7.5 mg of TZ/kg b.w.) dissolved in 1 mL of distilled water daily for 50 days.

The colors were administrated to non-fasted rats in between 9:00 and 10 a.m.

### 6.3. Collection of Samples

At the completion of the experiment, blood was taken out from the orbital sinus of fasting rats with a glass capillary according to Hassan et al. [55]. To obtain serum, the collected whole blood was relocated into non-heparinized centrifuge tubes made of glass materials and allowed to clot at room temperature and then centrifuged at 3500× g for 15 min. Blood serum was utilized for the lipid peroxide (LPO) and total antioxidant capacity (TAC) measurements. After being anesthetized, the rats were euthanized under mild anesthesia using diethyl ether. The liver and kidneys of the rats were taken out, washed in cold saline buffer, and then forwarded for analysis. Masses of individual kidneys and livers were determined, and the relative kidney and liver masses were calculated on the day of sacrifice based on the body masses measured.

### 6.4. Liver and Kidney Homogenates Preparation

Washed tissues samples were instantly snap frozen in liquid N_2_ and stored at −80 °C until further analysis. The enzyme homogenates were prepared using 1 g of fresh tissues (liver or kidney) homogenized in 10 mL of phosphate-buffered saline (PBS) 1:2 (*w/v*; 1 g tissue with 10 mL PBS, PH 7.4) for 15 min and centrifuged at 3000× *g* for 10 min to prepare. The centrifugated supernatant was transferred and used for the quantitation of oxidative stress biomarkers (reduced glutathione, GSH; total proteins, TP; and malonaldehyde, MDA) and antioxidant enzyme activities (superoxide dismutase, SOD; glutathione peroxidase, GPx; and catalase, CAT).

### 6.5. Lipid Peroxide (LPO) Levels Determination

The blood hydroperoxide level was assessed using an analytical system (Iram, Parma, Italy). The colorimetric test possesses the advantage of generating free radicals of hydroperoxide after reacting with transition metals. When a buffered chromogenic ingredient is added, a colored complex appears, which was measured spectrophotometrically.

The lipid peroxidation level (MDA) in the kidneys and liver homogenates was measured by the method of Ohkawa et al. [56] using thiobarbituric acid reacting substances (TBARS). Kidney and liver were homogenized in ice cold 10% Kcl (0.15 M) and the TBARS concentration was stated as nmol of MDA per mg protein utilizing 1,1,3,3-tetramethoxypropane as standard. The absorbance of the final material was recorded at 532 nm.

### 6.6. Determination of Total Antioxidant Capacity (TAC)

New generation acid radicals, colored (2,2′-azino-bis(3-ethylbenzothiazoline-6-sulfonic acid)) radical cation (ABTS^.+^) was applied for TAC determination. The ABTS^.+^ was decolorized by antioxidants with respect to their concentrations and antioxidant capacities. This change in color was recorded at 660 nm, the process was adopted to an automated analyzer, and the method was calibrated with Trolox as done by Ozcan Erel [57].

### 6.7. Determination of Reduced GSH of Liver and Kidney

Reduced glutathione was determined using the Ellman’s reagent, 5,5′-dithiobis(2-nitrobenzoic acid) (DTNB) as a coloring substance [58], and the absorbance of the colored matrial was recorded at 412 nm using a spectrophotometer (LKB-Pharmacia, Mark II, Ireland). Glutathione (GSH) concentration was determined from a standard calibration curve.

### 6.8. Analysis of Total Protein Concentration

The total protein concentrations in kidney and liver homogenates were assessed using a modified Lowry method discussed ealier by Schacter and Pollack [59], where the bovine plasma albumin was employed as standard.

### 6.9. Antioxidant Enzymes Activity

#### 6.9.1. SOD Activity

Superoxide dismutase (SOD, CuZnSO4, EC 1.15.1.1) activity in kidney and liver was analyzed spectrophotometrically following the method of Kakkar et al. [60]. The absorbance was recorded by setting the lambda max at 560 nm.

#### 6.9.2. CAT Activity

Catalase (CAT, EC1.11.1.6) activity in kidney and liver homogenates was determined by the reported method of Aebi [61] using H_2_O_2_ as the substrate. The disappearance of H_2_O_2_ was assayed at 240 nm and CAT activity was expressed as μmole^−1^min^−1^mg^−1^ protein.

#### 6.9.3. Estimation of GPx Activity

Glutathione peroxidase (EC 1.11.1.9) activity was assessed in kidney and liver homogenates following the method of Paglia and Valentine [62].

### 6.10. RNA Isolation

Frozen organ sections of the collected samples were grinded in a Mixer Mill of model MM 200 (Retsch GmbH and Co. KG, Haan, Germany) to a fine powder using pre-cooled stainless steel balls. Afterward, the total RNA was isolated using the total RNA Kit (iPrep™ PureLink™) equipped with an automated Purification Instrument (iPrep™, Invitrogen GmbH, Darmstadt, Germany), including a DNase digestion step as suggested by the manufacturers. The final material was quality controlled, adapting a gel analysis technique with the help of RNA HighSens Analysis Chips furnished with Bio-Rad Laboratories System (Experion, Munich, Germany). The only samples were used, which has a peak area ratio of 28S to 18S rRNA of ≥2.0. The RNA concentration levels were quantified with a Nano Drop 8000 Spectrophotometer manufactured by Thermo Scientific (Bonn, Gemrany).

### 6.11. Reverse-Transcriptase PCR

Quantitative reverse-transcriptase PCR (qRT-PCR) analysis was conducted for the expression of apoptosis-related genes following a previously reported method [63]. The synthesis of first-strand cDNA was achieved with 1 μg of total RNA using MLV Reverse Transcriptase and 100 ng of oligo-p(dT)12-18 primer with the help of Ready-To-Go RT-PCR Beads manufactured by GE Healthcare (GmbH, Germany). For preparing the internal housekeeping control, the expression levels were normalized to the GAPDH gene. A LightCycler^®^ 480 system with a 96-well plate (Roche Diagnostics) was employed for real-time quantification using the qPCR Green Master based on EvaGreen Fluorescent DNA Stain manufactured by Jena Bioscience (GmbH, Gemrany). The 20 μL final volume of PCR mixtures contained 5 μL of a 10:2 dilution of the cDNA, 10 μL of qPCR Green Master (Jena Bioscience) and primer, 300 nM each (Table 4).

The cycling conditions were involved in an initial heat-denaturing step (at 95 °C) with a Ramp Rate of 4.4 °C/s for the time period of 5 min, 50 cycles at 95 °C for 20 s at a Ramp Rate of 4.4 °C/s, annealing of all the primers at 60 °C for 15 s at a Ramp Rate of 2.2 °C/s, and signal acquisition (single mode) and product elongation at 72 °C for 15 s with a Ramp Rate of 4.4 °C/s. The melting curves were determined in continuous acquisition mode following the amplification in a three-segment cycle including 95 °C for 0 s, 65 °C for 15 s, and 95 °C for 0 s. The temperature transfer rates were set at 20 °C/s, while for segment three of the melting curve, the analysis was performed at 0.1 °C/s. Water was utilized as the template for amplifications of negative control included with each PCR analysis. Sequential dilutions of individual cDNA (10-1-10-6) were made to produce a quantitative standard curve of PCR, which was used to calculate the corresponding PCR efficiencies. Results were obtained from three biological replicates, while the PCR runs were repeated doubly.

### 6.12. qRT-PCR Fold Change Calculations

Roche LightCycler^®^ 480 Version 3.5 software was used for data analysis, while the Second Derivate Maximum technique was adapted to calculate the crossing point (CP). The target mRNA quantity was scrutinized and was normalized to the GAPDH gene mRNA. The 2−△△ Cp method was used to analyze the relative expression level (Fold change) [64]. Briefly, the calculation was accomplished using the equation:

Fold change (relative expression level) = 2 Cp target (control) − Cp target (treatment)/2 Cp GAPDH (control) − Cp GAPDH (treatment).(1)

The obtained fold changes data were examined by Student’s *t*-test to identify the important differences among dye-treated samples and untreated control. Treatments were considered statistically noteworthy when the value of *p* ≤ 0.05. Statistical methodology were performed using MINITAB software (State College, PA, USA, Version. 13.1, 2001).

### 6.13. Separation of Bone Marrow Cells

The separation o the bone mrrow cells was carried out following a previously described technique [65]. Briefly, femurs of both rats were dissectioned, taken out, and the muscles and other tissues were removed from them. A syringe filled with 1.5 mL of phosphate buffer saline (PBS) was used to flush the bone marrow cells and then centrifuged at 1200× *g* for 4 min. Cell pellets were washed with 1 mL of PBS twice to remove any PBS remainings. Afterwards, the cells were re-suspended in Ca^2+^ and Mg^2+^-free PBS (2 mL). A 500-μL aliquot of cell suspension was placed in four separate tubes for the comet assay analysis, intracellular ROS yields, and flow cytometric analysis to see the progression of the cell cycle and △Ψm. The adjustment of cells number were made to ≈1.0 × 10^6^ cells/mL for all the studied parameters. The determination of viability of cells, which were isolated from bone marrow, was made using trypan blue.

### 6.14. Comet Assay

The comet assay analysis of bone marrow cells was achieved following the in vivo comet assay guidelines described earlier [66]. In short, bone marrow cell suspension (100 μL) of vehicle (negative control), ethyl methanesulfonate (EMS)-treated (positive control), and TZ dye-treated rats were mixed with 1% low melting agarose (LMA) (100 μL). Then, the 80 μL cell suspension was layered onto one-third frosted slides previously coated with normal melting agarose (NMA) (1% in PBS) and stowed at 4 °C for 10 min. Then, a layer of LMA (0.5% in PBS) of 90 μL was added, and the cells were lysed in a lysing solution overnight. After that, the cells were taken out and washed with Milli Q water before being subjected to DNA denaturation at 4 °C for 20 min in cold electrophoretic buffer. Electrophoresis was carried out at 0.7 V/cm for 30 min at 4 °C applying a 300 mA (24 V) current. After that, the slides were washed thrice with neutralization buffer. To avoid any secondary DNA damage, the preparative steps were performed in darkness. Then, each and every slide was stained using ethidium bromide solution (75 μL) for 5 min. Fluorescence microscope (Nikon Eclipse 80i, Japan) was used at 40× magnification (excitation, 515–560 nm and emission, 590 nm), which was equipped with a charge-coupled device (CCD) camera. Then, 100 cell images (50 from each replicate slide) were randomly chosen, and the analysis of the image was achieved with software Comet Assay IV (Perceptive Instruments, Suffolk, UK). The means for the olive tail moment (OTM), tail intensity (%), and tail length (μm) were discretely analyzed for statistical significance. The statistical significance level was chosen *p* ≤ 0.05, unless otherwise specified.

### 6.15. Statistical Data Analysis

The statistical analysis and calculations were made using the Statistical Package for Social Sciences (SPSS) for Windows (Version 17.0). All the obtained data were represented as mean ± standard deviation (SD). Data were subjected to one-way analysis of variance (ANOVA) and Student’s *t*-test. Statistical probabilities of 0.01 were considered to be extremely important.

## 7. Conclusions

We have provided here evidence for the potential of Nano-CUR as a natural food coloring agent to minimize or prevent peroxidation, genotoxicity, and the expression of apoptosis-related genes, commonly taking place due to the consumption of various potentially hazardous foods and pharmaceutical drugs containing azo dyes by humans. Nano-CUR was found to delay the onset of the formation of potentially damaging ROS, eventually leading to a better capacity in maintaining nutritional food quality and more efficient cellular redox state homeostasis. This study has shown that Nano-CUR acting against oxidative stress and genotoxicity induced by TZ ingestion and the limitations of CUR (e.g., low stability, solubility, and bioavailability) can be overcome using Nano-CUR for the food, drug, and cosmetics industries.

## Figures and Tables

**Table 1:** Ameliorating effects of Nano-CUR on body weight, body weight gain, liver weight, kidney weight, relative liver weight, and relative kidney weights.

Parameters	G1	G2	G3	G4	G5
Initial Body Weight (g)	221.3 ± 1.73	225.9 ± 3.35	223.6 ± 2.51	224.6 ± 3.01	226.3 ± 3.32
Final Body Weight (g)	256.7 ± 3.31	211.5 * ± 3.41	261.8 ± 2.50	260.3 ± 2.52	262.2 ± 4.35
Body Weight Gain (g)	35.4 ± 0.32	−14.4 * ± 0.12	38.2 ± 0.34	35.7 ± 0.36	35.9 ± 0.38
Liver Weight (g)	5.00 ± 0.41	4.23 * ± 0.34	4.73 ± 0.33	5.51 ± 0.42	5.14 ± 0.34
Relative Liver Weight	1.95 ± 0.002	2.00 * ± 0.003	1.81 ± 0.001	1.98 ± 0.003	1.96 ± 0.004
Kidney Weight (g)	1.50 ± 0.04	0.59 * ± 0.06	1.25 ± 0.07	1.40 ± 0.07	1.48 ± 0.05
Relative Kidney Weight	0.58 ± 0.043	0.47 * ± 0.042	0.48 ± 0.041	0.54 ± 0.044	0.56 ± 0.051

Values are expressed as mean ± SD, * *p* < 0.05 relative to control (G1). G1 = Control, G2 = Tartrazine (TZ)-Ingested Rats, G3 = Nano-CUR-Ingested Rats, G4 = (1 g Nano-CUR + 7.5 mg TZ mix)-Ingested Rats, G5 = (2 g Nano-CUR + 7.5 mg TZ mix)-Ingested Rats.

**Table 2:** Effects of Nano-CUR on blood lipid peroxide (LPO) and total antioxidant capacity (TAC) as well as liver and kidney malondialdehyde (MDA) and reduced glutathione (GSH) in TZ-ingested rats.

Organs	Blood		Liver		Kidneys	
Parameters → Groups ↓	TAC mM/L	LPO mg/100 mL	MDA n mole/mg Protein	GSH n mole/mg Protein	MDA n mole/mg Protein	GSH n mole/mg Protein
G1	68.31 ± 4.21	29.31 ± 0.83	5.11 ± 0.08	354.3 ± 2.06	6.34 ± 0.05	348.3 ± 2.51
G2	45.62 ** ± 6.31	35.6 ** ± 4.21	6.63 ** ± 0.03	296.01 ** ± 4.31	8.1 ** ± 0.07	267.7 ** ± 4.21
G3	70.11 ± 2.31	28.6 ± 3.11	4.81 ± 0.07	355.6 ± 2.81	5.11 ± 0.02	352.1 ± 5.23
G4	65.63 ± 4.21	26.61 ± 5.31	5.61 ± 0.06	341.6 ± 4.21	6.72 ± 0.04	343.7 ± 2.20
G5	67.63 ± 4.16	25.21 ± 7.32	4.92 ± 0.03	350.0 ± 2.09	5.61 ± 0.04	351.3 ± 4.32

Values are expressed as means ± SD, ** *p* < 0.01 relative to control (G1), TAC = Total Antioxidant Capacity, LPO = Lipid Peroxide, MDA = Malonaldehyde, GSH = Glutathione Reduced. Group1 = Control, Group2 = TZ-treatment, Group3 = Nano-CUR treatment, Group4 = 1 (g) Nano-CUR + 7.5 mg TZ Mix. Treatment, Group5 = 2 (g) Nano-CUR + 7.5 mg TZ Mix. Treatment.

**Table 3:** Effect of Nano-CUR on total proteins and the activities of antioxidant enzymes in liver and kidney of TZ-treated rats.

Tissues	Liver				Kidney			
Parameters → Groups ↓	Proteins	SOD	CAT	GPx	Proteins	SOD	CAT	GPx
Group1	17.69 ± 1.35	7.06 ± 0.6	600.0 ± 6.36	185.6 ± 6.32	17.32 ± 1.36	6.21 ± 0.31	596.6 ± 7.39	179.6 ± 5.21
Group2	11.01 ** ± 0.99	4.63 ** ± 2.11	449.6 ** ± 2.11	122.3 ** ± 4.81	13.21 ** ± 1.11	4.16 ** ± 0.22	421.7 ** ± 9.21	120.1 ** ± 6.31
Group3	17.98 ± 1.33	7.21 ± 1.33	609.11 ± 3.92	186.2 ± 6.11	17.39 ± 1.93	6.71 ± 0.61	600.3 ± 6.82	180.6 ± 2.69
Group4	16.96 ± 2.31	6.71 ± 2.16	599.11 ± 2.83	181.3 ± 3.32	17.10 ± 2.21	5.82 ± 0.42	588.3 ± 8.29	178.6 ± 8.39
Group5	17.51 ± 2.12	6.98 ± 1.11	601.2 ± 3.61	185.3 ± 2.35	17.3 ± 3.29	6.11 ± 0.32	590.21 ± 6.33	180.1 ± 7.31

Values are expressed as means ± SD, ** *p* < 0.01 relative to control (G1), SOD = Superoxide Dismutase, CAT = Catalase, GPx = Glutathione Peroxidase. Group1 = Control, Group2 = TZ-treatment, Group3 = Nano-CUR treatment, Group4 = 1 (g) Nano-CUR + 7.5 mg TZ Mix Treatment, Group5 = 2 (g) Nano-CUR + 7.5 mg TZ Mix Treatment.

**Table 4:** Test of primers used for qRT-PCR expression analysis of apoptosis-related genes of rats.

Target Gene	Forward (F) and Reverse (R) Primers (5′-3′)	GenBank Accession No.	T(a), °C
Caspase-3	F: 5′-CAGAGCTGGACTGCGGTATTGA-3′ R: 5′-AGCATGGCGCAAAGTGACTG-3′	NM_012922	60
Caspase-9	F: 5′-AGCCAGATGCTGTCCCATAC-3′ R:5′-CAGGAGACAAAACCTGGGAA-3′	AF262319	60
Tp53	F: 5′-GTCGGCTCCGACTATACCACTATC-3′ R:5′-CTCTCTTTGCACTCCCTGGGGG-3′	NM_030989	60
GAPDH	F: 5′-GCTGCCTTCTCTTGTGACAAAGT-3′ R: 5′-CTCAGCCTTGACTGTGCCATT-3′	AF106860	60
